# Cellular Mechanisms of the Anti-Arrhythmic Effect of Cardiac PDE2 Overexpression

**DOI:** 10.3390/ijms22094816

**Published:** 2021-05-01

**Authors:** Michael Wagner, Mirna S. Sadek, Nataliya Dybkova, Fleur E. Mason, Johann Klehr, Rebecca Firneburg, Eleder Cachorro, Kurt Richter, Erik Klapproth, Stephan R. Kuenzel, Kristina Lorenz, Jordi Heijman, Dobromir Dobrev, Ali El-Armouche, Samuel Sossalla, Susanne Kämmerer

**Affiliations:** 1 Department of Pharmacology and Toxicology, Dresden University of Technology, 01307 Dresden, Germany; michael_wagner@tu-dresden.de (M.W.); mirna.sadeks@gmail.com (M.S.S.); johann.klehr@tu-dresden.de (J.K.); rebecca.firneburg@tu-dresden.de (R.F.); eleder.cachorro_puente@tu-dresden.de (E.C.); kurt.richter@outlook.de (K.R.); erik.klapproth@tu-dresden.de (E.K.); Stephan.kuenzel@tu-dresden.de (S.R.K.); 2 Klinik für Innere Medizin und Kardiologie, Dresden Heart Center, Dresden University of Technology, 01307 Dresden, Germany; 3 Clinic for Cardiology & Pneumology, University of Göttingen, 37075 Göttingen, Germany; ndybkov@med.uni-goettingen.de (N.D.); fleur.mason@med.uni-goettingen.de (F.E.M.); samuel.sossalla@ukr.de (S.S.); 4 DZHK (German Centre for Cardiovascular Research), 10785 Berlin, Germany; 5 Department of Pharmacology and Toxicology, Julius-Maximilians-Universität Würzburg, 97078 Würzburg, Germany; lorenz@toxi.uni-wuerzburg.de; 6 Leibniz-Institut für Analytische Wissenschaften-ISAS-e.V., 44139 Dortmund, Germany; 7 Department of Cardiology, CARIM School for Cardiovascular Diseases, Faculty of Health, Medicine, and Life Sciences, Maastricht University, 6200 MD Maastricht, The Netherlands; Jordi.heijman@maastrichtuniversity.nl; 8 Institute of Pharmacology, West German Heart and Vascular Center, University Duisburg-Essen, 45147 Essen, Germany; Dobromir.dobrev@uk-essen.de; 9 Montréal Heart Institute, Research Center, University de Montréal, Montréal, QC H1T 1C8, Canada; 10 Department of Molecular Physiology Biophysics, Baylor College of Medicine, Houston, TX 77030, USA; 11 Department of Internal Medicine II, University Hospital Regensburg, 93042 Regensburg, Germany

**Keywords:** PDE2, arrhythmia, CaMKII, heart failure

## Abstract

Background: Phosphodiesterases (PDE) critically regulate myocardial cAMP and cGMP levels. PDE2 is stimulated by cGMP to hydrolyze cAMP, mediating a negative crosstalk between both pathways. PDE2 upregulation in heart failure contributes to desensitization to β-adrenergic overstimulation. After isoprenaline (ISO) injections, PDE2 overexpressing mice (PDE2 OE) were protected against ventricular arrhythmia. Here, we investigate the mechanisms underlying the effects of PDE2 OE on susceptibility to arrhythmias. Methods: Cellular arrhythmia, ion currents, and Ca^2+^-sparks were assessed in ventricular cardiomyocytes from PDE2 OE and WT littermates. Results: Under basal conditions, action potential (AP) morphology were similar in PDE2 OE and WT. ISO stimulation significantly increased the incidence of afterdepolarizations and spontaneous APs in WT, which was markedly reduced in PDE2 OE. The ISO-induced increase in I_CaL_ seen in WT was prevented in PDE2 OE. Moreover, the ISO-induced, Epac- and CaMKII-dependent increase in I_NaL_ and Ca^2+^-spark frequency was blunted in PDE2 OE, while the effect of direct Epac activation was similar in both groups. Finally, PDE2 inhibition facilitated arrhythmic events in *ex vivo* perfused WT hearts after reperfusion injury. Conclusion: Higher PDE2 abundance protects against ISO-induced cardiac arrhythmia by preventing the Epac- and CaMKII-mediated increases of cellular triggers. Thus, activating myocardial PDE2 may represent a novel intracellular anti-arrhythmic therapeutic strategy in HF.

## 1. Introduction

Heart failure (HF) remains one of the leading causes of mortality worldwide. Patients with HF are at risk for lethal ventricular arrhythmias due to structural and electrical remodeling [1]. Beyond various complex mechanisms, chronically stimulated cyclic adenosine monophosphate (cAMP) pathways upon sympathetic activation contribute to generation of arrhythmia [2,3]. Arrhythmia-related sudden cardiac death (SCD) accounts for up to 60% of deaths in HF patients [4]. Therefore, innovative antiarrhythmic therapeutic concepts are highly desired. In contrast to cAMP, the levels of cyclic guanosine monophosphate (cGMP) are reduced in many HF patients [5,6]. Strategies aiming to enhance cGMP signaling have been considered beneficial for HF patients [7,8,9].

Regular heart rhythm and contraction require a coordinated action potential (AP) generation followed by coordinated intracellular Ca^2+^ cycling coupling the excitation-contraction processes [10]. Cellular depolarization upon sodium influx (I_Na_) through voltage-gated Nav1.5 channels triggers the opening of voltage-gated L-type Ca^2+^ channels, Cav1.2 (LTCC). The resulting L-type calcium current (I_CaL_) subsequently initiates the activation of ryanodine receptors (RyR2), allowing Ca^2+^ release from the sarcoplasmic reticulum (SR) into the cytosol which finally evokes myofilament contraction. Cardiomyocyte relaxation is mainly mediated by SR Ca^2+^-ATPase type-2a (SERCA2a), which pumps cytosolic Ca^2+^ back to the SR. Potassium extrusion from the myocyte via different K^+^ channels, generating the transient outward current (I_to_), the delayed rectifier (I_Ks_, I_Kr_), and inward rectifier (I_K1_) currents, contributes to repolarization thereby restoring the resting membrane potential.

The activity of ion channels, as well as Ca^2+^ cycling proteins, is modulated by the cAMP-dependent protein kinase A (PKA) as well as Ca^2+^-calmodulin-dependent protein kinase II (CaMKII) upon β-adrenergic stimulation [11,12,13,14,15,16]. Both kinases affect Ca^2+^ cycling proteins such as RyR2 and the SERCA2a-inhibiting phospholamban (PLB) facilitating SR Ca^2+^ release and reuptake. CaMKII and PKA have been known to influence ion channel proteins like Nav1.5, Cav1.2, and Kv4 [12,13,14,15,16]. Impaired phosphorylation of these proteins promotes cellular pro-arrhythmic events. Thus, the increase in late I_NaL_, I_CaL_, and Ca^2+^ leaks from the SR via RyR2 have been shown to prolong AP duration and trigger early (EAD) and delayed afterdepolarization (DAD), as well as spontaneous APs (sAP) promoting the development of ventricular fibrillation associated with SCD [11,17].

Phosphodiesterases (PDE) play a key role in regulating myocardial cAMP and cGMP levels. Among the family of cardiac PDEs, phosphodiesterase 2 (PDE2) has the unique property to be stimulated by cGMP via its GAF domains to primarily hydrolyze cAMP [18]. Thus, PDE2 can mediate a negative crosstalk between cGMP and cAMP signaling pathways [19]. The enzyme exists in three isoforms PDE2A1, 2, and 3, which are all generated by alternative exon splicing [19,20]. PDE2 isoforms are localized within the cytoplasm (mainly PDE2A1) or the membrane fractions comprising the plasma and nuclear membrane, sarcoplasmic reticulum, and the Golgi body (mostly PDE2A3) [21,22]. The PDE2A2 isoform has been shown to regulate local mitochondria-related cAMP pools [23,24,25]. In cardiomyocytes, basal cAMP-hydrolytic activity of PDE2 is rather low but increases substantially under β-adrenergic stimulation and pathophysiological conditions [21,26,27]. In contrast to other cardiac PDEs, PDE2 is upregulated in human as well as in experimental HF models, where it contributes to the desensitization process to β-adrenergic overstimulation [28].

In previous work, we studied the effects of chronically increased PDE2 activity in the heart using the transgenic mouse line with cardiac-specific overexpression of the wild-type murine PDE2A3 isoform (PDE2 OE, see Methods). We observed that PDE2 OE mice showed lower susceptibility to arrhythmia after acute provocation by double isoprenaline (ISO) injections, displaying less premature ventricular contractions and ventricular tachycardia than control mice (WT) [29]. Under pathological conditions of ligation of the left anterior descending coronary artery (LAD) for two weeks, PDE2 OE mice showed a significantly higher ejection fraction compared to WT mice. Moreover, PDE2 OE mice displayed a survival benefit due to protection from lethal arrhythmia [29].

In this study, we focused on investigating the role of PDE2 concerning the susceptibility to cardiac arrhythmias and on deciphering the underlying cellular mechanisms. We reveal that increased PDE2 activity protects against ISO-induced cellular arrhythmic events. Blunting of the ISO-induced, ‘exchange protein directly activated by cAMP’ (Epac) and CaMKII-mediated increases in I_NaL_, I_CaL_, and SR Ca^2+^ leak, were identified as underlying mechanisms. Thus, cGMP-mediated activation of myocardial PDE2 may represent a novel intracellular anti-arrhythmic therapeutic strategy in HF.

## 2. Results

**Figure 1:**
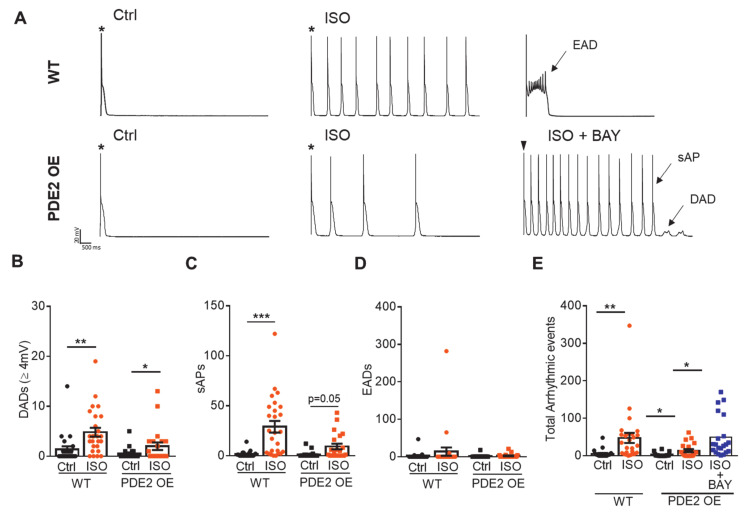
PDE2 OE display diminished cellular arrhythmogenic events. (**A**) Representative traces recorded in whole-cell patch clamp mode from WT (upper) and PDE2 OE (lower) ventricular cardiomyocytes upon stimulation at 0.125 Hz following stimulation at 4 Hz. Stars represent the stimulated action potentials; the following signals are spontaneous. Measurements were performed under basal conditions (Ctrl, black symbols) and upon β-adrenergic stimulation with isoprenaline (ISO, 100 nM, 10 min, orange symbols) and simultaneous PDE2 inhibition with BAY60-7550 (BAY, 100 nM). Quantification of (**B**) delayed afterdepolarizations (DAD, ≥4 mV), (**C**) spontaneous action potentials (sAP), (**D**) early afterdepolarizations (EAD), and (**E**) total number of arrhythmic events per cell in WT (circles) and PDE2 OE (squares). * *p* < 0.05, ** *p* < 0.01, *** *p* < 0.001 (N = 8 animals/genotype, 20 ≤ n ≤ 26 cells/group).

Mice with cardiac-specific PDE2 overexpression (PDE2 OE) displayed significantly fewer arrhythmic events upon β-adrenergic stress triggered by isoprenaline (ISO) injections, as well as after myocardial infarction via left anterior descending coronary artery (LAD) ligation [29]. In order to study the potential antiarrhythmic effect of PDE2 overexpression at the cellular level, we quantified early and delayed afterdepolarizations (EAD, DAD) and spontaneous action potentials (sAP) in isolated cardiomyocytes from PDE2 OE mice and respective wildtype (WT) littermates. Figure 1A depicts representative action potential (AP) recordings in WT and PDE2 OE at basal conditions (Ctrl) and upon isoprenaline application (ISO, 100 nM, 10 min) at 0.125 Hz after stimulating the cardiomyocyte at 4 Hz. Under physiological conditions, the number of occurred DADs, EADs, and sAPs did not differ between WT and PDE2 OE cells (Figure 1B-D). β-adrenoceptor stimulation with ISO significantly increased the number of DADs and sAPs in WT, but not in PDE2 OE (Figure 1B,C). The number of EADs also tended to be higher in WT than OE (Figure 1D). The total number of arrhythmogenic events upon ISO was significantly lower in cardiomyocytes from PDE2 OE compared to WT (Figure 1E). Specific inhibition of PDE2 with BAY 60-7550 (BAY, 100 nM) increased ISO-induced arrhythmogenic events in PDE2 OE cells to the level of WT (Figure 1E). Thus, the antiarrhythmic effect of PDE2 overexpression under β-adrenergic stimulation was also observed at cardiomyocyte level.

**Figure 2:**
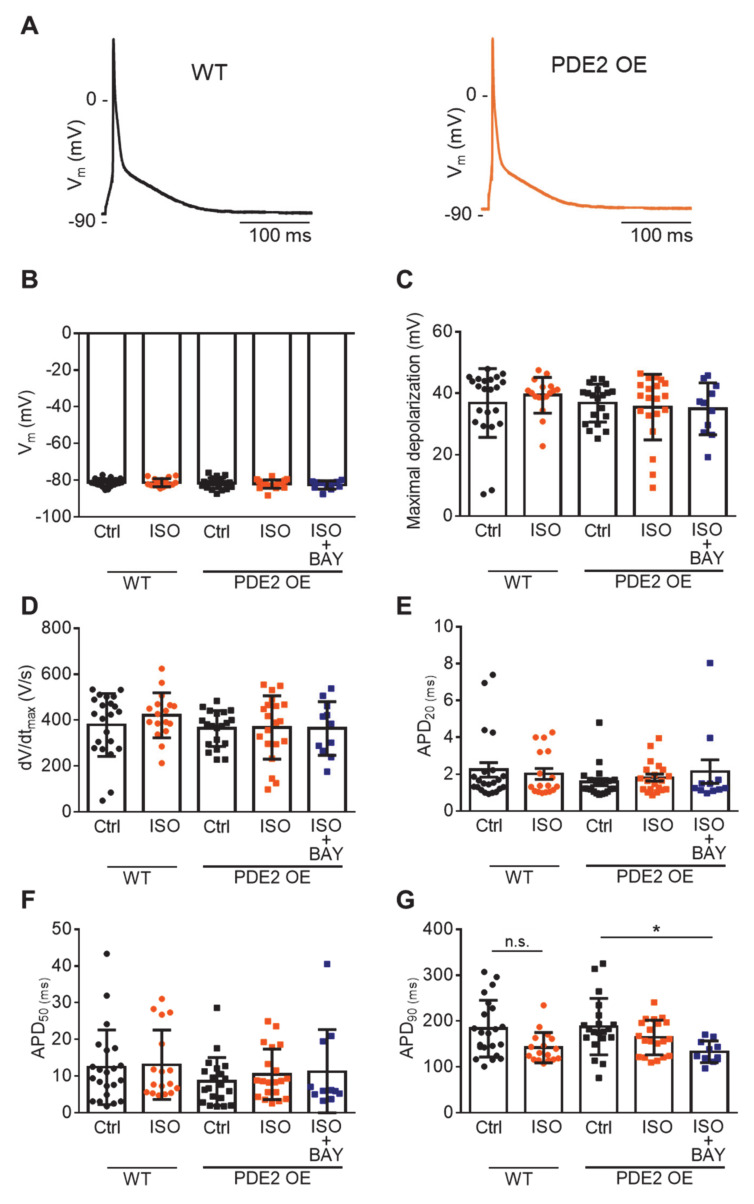
Similar basal action potential properties in PDE2 OE and WT. (**A**) Representative voltage traces of action potentials (AP) at 1 Hz from left ventricular myocytes of WT (circles) and PDE2 OE (squares) mice under basal conditions (Ctrl, black symbols) and upon β-adrenergic stimulation with isoprenaline (ISO, 100 nM, 10 min, orange symbols) and simultaneous PDE2 inhibition with BAY60-7550 (BAY, 100 nM, dark blue symbols). (**B**) Average resting membrane potential V_m_ (mV), (**C**) maximum depolarization potential (mV), (**D**) maximal upstroke velocity dV/dt (V/s), (**E**–**G**) action potential duration at 20%, 50%, and 90% repolarization (APD_20_, APD_50_, APD_90_). n.s. not significant, * *p* < 0.05 (N = 8 animals/genotype, 11≤ n ≤ 22 cells/group).

Representative AP recordings in cardiomyocytes from WT and PDE2 OE mice under basal conditions and ISO stimulation (100 nM, 10 min) at 1 Hz from the same series of experiments are depicted in Figure 2A. PDE2 overexpression did not significantly affect the resting membrane potential, AP depolarization, and repolarization phase under physiological conditions, as well as β-adrenergic stimulation (Figure 2B–G). However, there was a tendency towards reduced AP duration at 20% and 50% repolarization (APD_20_, APD_50_) in PDE2 OE compared to WT. Conversely, upon ISO stimulation, there was a trend towards increased APD_90_ (Figure 2E–G).

To evaluate the cellular mechanisms of antiarrhythmic PDE2 effects, we studied intracellular Ca^2+^-handling in cardiomyocytes of PDE2 OE and WT littermates. First, L-type calcium current (I_CaL_) was measured under basal conditions and upon ISO stimulation (Figure S1A, [30]). PDE2 OE cells displayed similar I_CaL_ voltage-membrane (IV) relationship as WT cardiomyocytes under basal conditions (Figure S1B). However, the increase in I_CaL_ upon β-adrenergic stimulation was suppressed in OE cells. In WT cells, I_CaL_ significantly increased upon ISO stimulation in a concentration-dependent manner showing the maximum amplitude at 100 nM ISO. In contrast, PDE2 overexpression prevented ISO-induced increase in I_CaL_ even at the highest concentrations tested (Figure S1C,D) [29].

**Figure 3:**
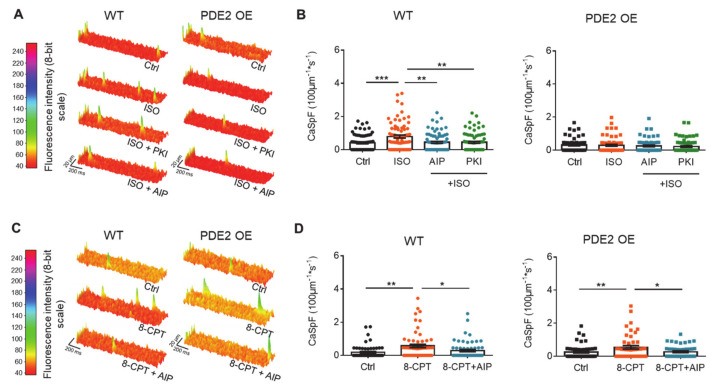
β-AR stimulation does not increase Calciumspark frequency upon β-adrenergic stimulation in PDE2 OE. Ventricular myocytes were loaded with fluo-4 AM and monitored for spontaneous intracellular rises in Ca^2+^ levels (Ca^2+^ sparks) by line scan confocal microscopy (**A**,**B**) under basal conditions (black) and after 10 min stimulation with ISO (100 nM, orange), ISO (100 nM) and AIP (1 μmol/L, blue), ISO (100 nM) and PKI (5 μmol/L, green); (**C**,**D**) 8-CPT (10 μmol/L, orange), 8-CPT (10 μmol/L) and AIP (1 μmol/L, blue). (**A**,**C**) Representative original registrations and (**B**,**D**) quantification of Ca^2+^ spark frequency (CaSpF) normalized to cell width and scan rate from WT (circles) and PDE2 OE (squares). * *p* < 0.05, ** *p* < 0.01 *** *p* < 0.001. (N = 4 animals/genotype, 45 ≤ n ≤ 79 cells/group).

In the next step, spontaneous intracellular Ca^2+^ release was quantified because Ca^2+^ sparks can provoke DAD events. Under basal conditions, PDE2 OE displayed similar calcium spark frequency (CaSpF) as cardiomyocytes from WT littermates (Figure 3A,B). Upon β-adrenergic stimulation with ISO (100 nM), the increase in CaSpF seen in WT was not observed in PDE2 OE. Simultaneous inhibition of the kinases CaMKII and PKA using Autocamptide-2 Related Inhibitor Peptide (AIP, 1 µM) or PKI (5 µM), respectively, prevented the ISO-induced increase in CaSpF in WT cells. In contrast, ISO stimulation with or without inhibition of CaMKII or PKA had no effect on CaSpF in OE mice (Figure 3A,B). To ensure the function of CaMKII, the direct activator 8-CPT of the ‘exchange protein directly activated by cAMP’ (Epac) was used to stimulate CaMKII activity. 8-CPT (10 µM) stimulation significantly increased CaSpF to the same extent in cardiomyocytes from PDE2 OE and WT, revealing a comparable CaMKII action in both genotypes (Figure 3C,D).

**Figure 4:**
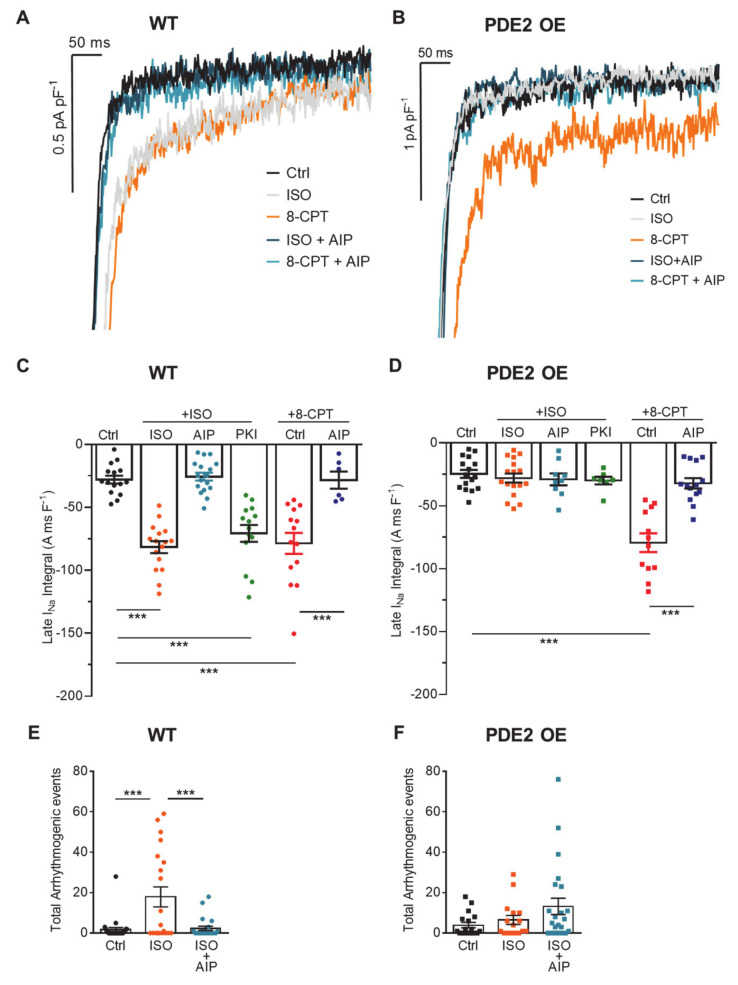
Ca^2+^/calmodulin-dependent kinase II (CaMKII)-mediated increase in I_NaL_ and cellular arrhythmogenic events upon β-AR stimulation is abolished in PDE2 OE. Late sodium current (I_NaL_) was measured in isolated ventricular cardiomyocytes from PDE2 OE (squares) and WT (circles) mice at −35 mV (1 s) under basal conditions (black symbols) and after 10 min stimulation with ISO (100 nM, orange), ISO (100 nM) and AIP (1 µM, blue), ISO (100 nM) and PKI (5 µM, green), 8-CPT (10 µM, red), 8-CPT (10 µM) and AIP (1 µM, dark blue). (**A**,**B**) Representative current traces and (**C**,**D**) quantification of I_NaL_ estimated by integrating I_Na_ between 100 and 500 ms and normalized to the membrane capacitance. *** *p* < 0.001 vs. control (Ctrl), (N = animals/genotype, 6 ≤ n ≤ 17 cells per group). (**E**,**F**) Isolated ventricular cardiomyocytes were subjected to an arrhythmia provocation protocol under basal conditions (black), after 10 min stimulation with ISO (100 nM, orange) or ISO (100 nM) and AIP (1 µM, blue). Arrhythmogenic events (DADs, EADs, and sAPs) were quantified. *** *p* < 0.001; 8≤ n ≤ 13, (N = 5 animals/group).

It has previously been shown that increasing the late sodium current (I_NaL_) can promote the generation of arrhythmia by AP prolongation and initiation of early afterdepolarizations [31]. Therefore, we investigated the effect of PDE2 overexpression on I_NaL_ amplitude upon β-adrenergic stimulation. Under basal conditions, I_NaL_ was similar in cardiomyocytes of PDE2 OE and WT mice. ISO incubation (100 nM, 10 min) significantly enhanced I_NaL_ in WT cells whereas this effect was not present in PDE2 OE (Figure 4A–D). The inhibition of CaMKII with AIP (1 µM) prevented the I_NaL_ elevation upon β-adrenergic stimulation in WT. In contrast, PKA inhibition with Protein kinase inhibitor peptide (PKI, 5 µM) did not affect the ISO-mediated I_NaL_ increase (Figure 4A–C) indicating a major contribution of CaMKII to I_NaL_ elevation. However, in both groups the stimulation of CaMKII via the Epac activator 8-CPT clearly resulted in enhanced I_NaL_ to comparable levels as that induced by ISO in WT (Figure 4C,D). CaMKII inhibition significantly reduced the 8-CPT-induced increase in I_NaL_ in both groups. To test the influence of CaMKII on arrhythmogenic events at the cellular level, ISO-induced arrhythmogenic events including DADs, EADs, and sAPs were quantified after CaMKII inhibition with AIP. Indeed, in WT cells AIP significantly lowered the number of ISO-induced arrhythmogenic events. In PDE2 overexpressing cells, the ISO-induced increase in cellular arrhythmia was blunted showing no effect of CaMKII inhibition (Figure 4E,F).

**Figure 5:**
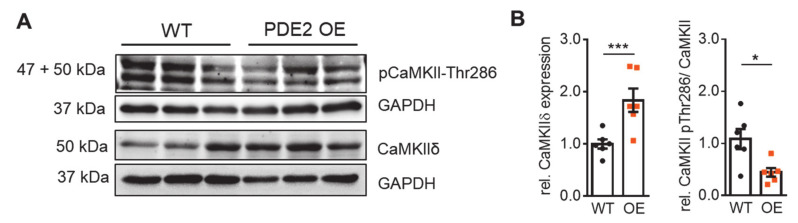
Basal expression and phosphorylation of CaMKII is affected by PDE2 overexpression. (**A**) Representative immunoblots and (**B**) quantification of protein kinase expression normalized to GAPDH, CaMKII phosphorylation at Threonine 286 normalized to total CaMKIIδ in lysates of isolated cardiomyocytes from PDE2 OE (orange) and WT (black) mice. (N = 6 animals/genotype. * *p* < 0.05, *** *p* < 0.001 vs. WT).

PDE2 modulates the activity of downstream protein kinases via hydrolysis of ISO-induced cAMP. Besides direct activation of PKA, cAMP-dependent activation of Epac stimulates phospholipase C (PLC), which in turn hydrolyzes phosphatidylinositol 4,5-bisphosphate (PIP_2_) to produce diacylglycerol (DAG) and inositol triphosphate (IP_3_) leading to PKC and CaMKII activation [32]. These kinases contribute to regulation of contractile protein phosphorylation and myofilament Ca^2+^ sensitivity. Therefore, we evaluated the consequences of PDE2 overexpression on expression levels of selected cardiac kinases in lysates of isolated ventricular cardiomyocytes (Figure 5, Supplemental Figure S2). Western blots did not reveal evidence for significant regulation of PKC, PKA-C, and its regulatory subunit PKAIIα, as well as Epac protein levels in PDE2 OE compared to WT (Supplemental Figure S2A,B). Interestingly, under basal conditions the expression of CaMKIIδ was significantly increased in PDE2 OE whereas the phosphorylation of CaMKII at Threonine 286 was significantly reduced in PDE2 overexpressing cells contributing to diminished β-adrenergic response in PDE2 OE with respect to WT (Figure 5).

In previous work, we studied the consequences of PDE2 overexpression on cardiac Ca^2+^ cycling proteins showing reduced phosphorylation levels of RyR2 and PLB at CaMKII-specific phosphorylation sites in PDE2 OE but similar protein expression compared to WT [29]. Here, we also quantified the protein levels of the Na^+^/Ca^2+^ exchanger type 1 (NCX1), Na^+^/K^+^ ATPase (NKA), and phospholemman (PLM), which are important for the maintenance of intracellular Na^+^ and Ca^2+^ homeostasis. Protein levels of NCX1, NKA, and PLM did not differ between PDE2 OE and WTThe phosphorylation level of PLM at Ser68 was similar in both genotypes (Supplemental Figure S2C,D).

In order to evaluate whether our experimental data obtained in mice can be translated to large mammals, we employed a recent computational model of the canine ventricular cardiomyocyte with localized cAMP and CaMKII signaling [33]. In consistency with our experimental data, a simulated two-fold global PDE2 overexpression decreased cAMP levels, CaMKII activity, as well as PKA-dependent and CaMKII-dependent phosphorylation of L-type Ca^2+^ channels, ryanodine receptor 2 (RyR, Figure S3), and other targets, whereas 10-fold PDE2 overexpression completely prevented ISO-induced phosphorylation of all electrophysiological targets (not shown). Similarly, there was a reduction in I_CaL_, I_NaL_, and whole-cell Ca^2+^ transient in the presence of two-fold PDE2 overexpression (Figure S4), providing initial evidence that the protective mechanisms identified here may also apply in large mammals.

Altered potassium currents contribute to development of cardiac arrhythmia by delaying membrane repolarization and prolonging the AP duration [34]. Therefore, potassium outward currents were studied in cardiomyocytes from PDE2 OE and WT. Quantification of different current components revealed that PDE2 overexpression did not affect the fast transient outward K^+^ current (I_to_) or the sustained K^+^ current component (I_sus_). Elevated K^+^ current densities of the slow component (I_K,slow_) were observed in PDE2 OE at membrane potentials above +40 mV compared to WT (Figure S5). Furthermore, the inwardly rectifying K^+^ current (I_K1_) was significantly reduced in PDE2 OE at membrane potentials below −100 mV, compared to WT (Figure S5).

**Figure 6:**
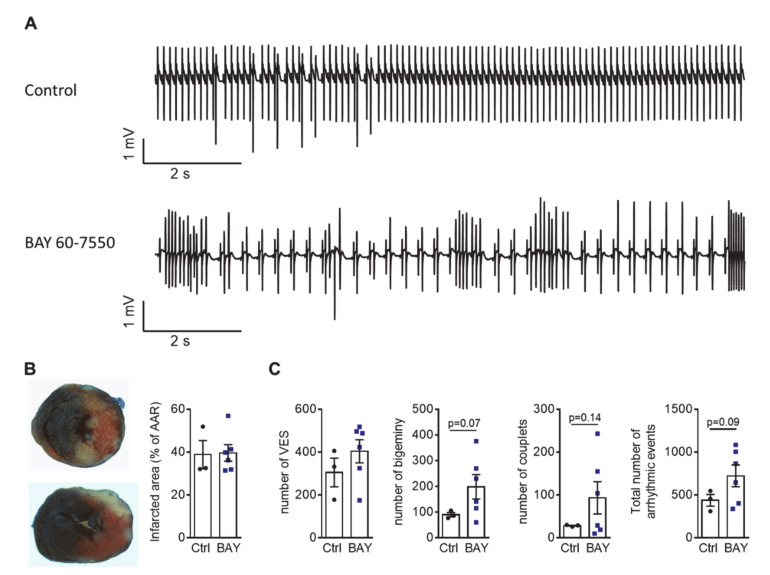
Specific PDE2 inhibition tends to increase the number of total arrhythmic events in *ex vivo* perfused hearts after reperfusion injury. (**A**) Representative traces of ECG recordings after reperfusion injury with 30 min of LAD ligation in *ex vivo* perfused hearts with and without PDE2 inhibitor BAY 60-7550 (BAY, 100 nM). (**B**) Evans blue and TTC staining of heart slices after reperfusion injury and quantification of infarcted area at risk (AAR, %). (**C**) Quantification of ventricular extrasystoles (VES), bigeminy, couplets and total number of arrhythmic events. Statistical *p*-values vs. Ctrl as indicated (N = 3–6 hearts/group).

Finally, the influence of PDE2 on arrhythmia development was studied at organ level in *ex vivo* perfused WT hearts during reperfusion after 30 min of ischemia via ligation of the left ascending artery using Krebs–Henseleit buffer with physiological catecholamine concentrations of 10 nM norepinephrine and 3.5 nM epinephrine (Figure 6A). The infarcted areas were similar in hearts perfused with the PDE2 inhibitor BAY 60-7550 (100 nM) and control hearts (Figure 6B). However, electrocardiogram (ECG) measurements revealed that PDE2 inhibition enhanced the number of cardiac arrhythmic events, such as ventricular extrasystoles (VES), premature ventricular complexes (PVC, bigeminy, couplets, triplets), and ventricular tachycardia, after reperfusion injury, compared to control hearts (Figure 6C).

## 3. Discussion

PDE2 overexpression (PDE2 OE) has previously been shown to provide marked protection against catecholamine induced ventricular arrhythmia under basal conditions, as well as after myocardial infarction (MI), leading to improved survival after MI [29]. In this study, we deciphered the underlying mechanisms of antiarrhythmic PDE2 functions at the cellular level.

Triggered cardiac arrhythmias are associated with frequent occurrence of early and delayed afterdepolarizations (EAD, DAD), as well as spontaneous action potentials (sAP), triggering inhomogeneous excitation, reentry and ventricular tachycardia [11]. Indeed, cardiac-specific PDE2 overexpression exhibited a lower susceptibility to cellular EAD, DAD, and sAP after β-adrenergic stimulation compared to WT. Inhibition of PDE2 activity in cells of transgenic mice markedly increased the number of arrhythmogenic events confirming the protective effect of PDE2. Thereby, augmented PDE2 activity did not significantly affect action potential (AP) morphology under basal conditions. The underlying currents were similar between PDE2 OE and wildtype (WT), which showed comparable I_to_, I_CaL_, I_NaL_, and I_K1_ amplitudes at physiological membrane potentials. In murine cardiomyocytes, under basal conditions PDE2 accounts only for ~3% of the total cAMP hydrolytic activity [21,28]. Thus, physiological cardiac excitation is not influenced by PDE2 activity at basal levels.

In contrast to other PDEs, PDE2 activity is increased upon chronic β-adrenergic stimulation [28,35]. Under chronic cardiac stress conditions, PDE2 is upregulated, as has been detected in human heart failure as well as in animal models after chronic β-AR stimulation, rapid pacing, or pressure overload [18,28]. Thereby, PDE2 upregulation is a direct consequence of β-adrenergic overstimulation. In another study, cardiomyocytes from rats chronically treated with isoprenaline (ISO), PDE2 inhibition revealed a marked increase in cAMP, whereas the SNP-cGMP-dependent stimulation of PDE2 resulted in significantly reduced cAMP levels in ISO-treated cells in contrast to control myocytes [28]. In a human failing heart, cAMP levels are reduced due to desensitized β-adrenergic signal transduction [36]. However, clinical trials with PDE3 inhibitors to normalize cardiac cAMP levels in HF patients have shown increased mortality due to proarrhythmic effects promoting sudden cardiac death [37,38]. Thus, under β-adrenergic stress conditions, enhanced PDE2 activity might serve to modulate cAMP-dependent protein activity underlying cardiac excitation and thus reducing arrhythmogenic cellular events.

Voltage-gated Na^+^ and L-type Ca^2+^ channels play a pivotal role in the initiation and propagation of the action potential in cardiomyocytes. In heart failure, impaired inactivation of Na^+^ channels induces a sustained Na^+^ current (late I_Na_) [39]. Higher intracellular Na^+^ concentration caused by increased I_NaL_ could lead to increased sarcoplasmic reticulum (SR) Ca^2+^ content via altered Na^+^/Ca^2+^ exchanger activity, thus promoting spontaneous SR Ca^2+^ release, which can trigger arrhythmia. Furthermore, increased I_NaL_ and I_CaL_ promote AP prolongation in heart failure (HF), increasing the susceptibility to arrhythmia generation [40]. The existence of a tight microdomain among β-ARs, L-type calcium channels and PDE2 has been reported in atrial, as well as ventricular cardiomyocytes [17]. In the present study, we showed that PDE2 overexpression prevented the ISO-induced increase in I_Ca,L_, I_NaL_, and spontaneous SR Ca^2+^ sparks. The reduction in these cellular pro-arrhythmic triggers contributes to reduced DADs and sAPs at cellular level (this study) and the development of arrhythmia *in vivo* [29]. Accordingly, *ex vivo* heart perfusion with BAY 60-7550 provoked increased incidence of arrhythmia. Further studies are needed to study the effects of PDE2 on the function of the Na^+^/Ca^2+^ exchanger and Na^+^/K^+^ ATPase (NKA), because they are important for maintaining intracellular Na^+^ and, consequently, regulation of the Na^+^/Ca^2+^ exchanger and Ca^2+^ homeostasis [41].

It has been demonstrated that PDE2 affects L-type calcium currents in mice, but that its modulation is different in atrial versus ventricular cardiomyocytes. In human and murine atrial myocytes, PDE2 inhibition with erythro-9-(2-hydroxy-3-nonyl)adenine (EHNA) resulted in stimulation of I_CaL_ under basal conditions, whereas no effect on I_CaL_ was observed in murine ventricular myocytes [42,43]. Accordingly, here we have shown that PDE2 overexpression does not affect basal I_CaL_ current density in ventricular cardiomyocytes. In rat atria, PDE2 inhibition with BAY 60-7550 increased basal beating but did not affect β-AR-induced sinoatrial tachycardia [44]. Furthermore, *in vivo* administration of BAY 60-7550 (i.p.) significantly increased heart rate in dogs and rats [28]. Correspondingly, PDE2 OE mice displayed lower heart rates, but preserved cardiac function [29]. Further studies are needed to reveal cellular PDE2 effects on ion currents in sinoatrial node and atrial cardiomyocytes.

The observed antiarrhythmic PDE2 effects were mainly mediated by reduced activity of the cAMP-dependent Epac/CaMKII axis. While basal expression of PKA was similar in PDE2 OE and WT mice, CaMKII expression was significantly increased in PDE2 OE, potentially due to adaptive upregulation with respect to increased cAMP hydrolysis upon β-adrenergic stimulation. In addition, CaMKII phosphorylation was clearly reduced at basal conditions, suggesting a PDE2 association with CaMKII in defined subcellular pools. Thus, pharmacological inhibition of CaMKII (and PKA) did not affect the blunted ISO-induced increase in I_NaL_ and SR Ca^2+^ leak in PDE2 OE. The cAMP-independent CaMKII stimulation via direct activation of Epac enhanced the I_NaL_ and Ca^2+^ spark frequency in PDE2 overexpressing cells confirming the physiological action of Epac/CaMKII in PDE2 OE. In HF, pathological CaMKII signaling promotes cardiac arrhythmia by influencing ion channel activities including I_Na_, I_CaL_ and various potassium currents [45]. Especially, CaMKII facilitates I_NaL_, as well as SR Ca^2+^ leak from RyR2 via phosphorylation in HF, thereby provoking arrhythmia [31,46]. It has been shown that β-AR-mediated CaMKII activation via β-arrestin-CaMKII-Epac complex induces SR Ca^2+^ leak via phosphorylation of RyR2-Ser2814 [47]. An increase in CaMKII auto-phosphorylation due to directly activated Epac (by 8-CPT) leads to enhancement of I_NaL_ [48]. Hence, blunted β-AR-mediated increase in I_NaL_ observed in PDE2 OE was specifically mediated via the Epac/CaMKII pathway, but not by PKA. In previous work, we could show that the steady-state phosphorylation levels of RyR2 at its CaMKII phosphorylation site Ser2814 were significantly lower in PDE2 OE, whereas the status at its PKA-phosphorylation site Ser2808 was not altered [29]. In addition, phosphorylation of PLB at Ser16 (PKA) and Thr17 (CaMKII) were both decreased in tissue lysates from PDE2 OE compared to WT. The basal protein levels of the key Ca^2+^-handling proteins RyR2, PLB, and SERCA2a were similar between PDE2 OE and WT. Although basal Ca^2+^ transient amplitude and SR Ca^2+^ load did not differ between PDE2 OE and WT, the ISO-related increase in Ca^2+^ transient amplitude was lower in cardiomyocytes from PDE2 OE [29].

Other studies have revealed PDE2 regulation of a local cAMP pool that acts anti-hypertrophically by activating PKAII, leading to phosphorylation of nuclear factors of activated T cells (NFAT) and reducing nuclear localization, which reduces the expression of hypertrophic genes [49]. Furthermore, chronic pharmacological PDE2 inhibition with BAY 60-7550 preferentially promoted NO/guanylyl cyclase/cGMP signaling and reversed the development of hypertrophy [50]. Thus, the conflicting evidence suggests that PDE2 may have protective anti-arrhythmic, as well as detrimental pro-hypertrophic effects in the cardiomyocyte, dependent on isoform, localization, and cellular and pathophysiological context.

The limitation of potential PDE2 spillover should be carefully considered. Artificial PDE2A3 expression within subcellular compartments cannot be excluded. Our in silico analysis suggests that PDE2 overexpression may produce similar changes in cardiomyocyte calcium handling in large mammals as we observed experimentally in mice. However, at present there are no *in silico* models incorporating detailed cAMP and CaMKII signaling that can also simulate spontaneous SR calcium-release events and afterdepolarizations, precluding direct validation of the antiarrhythmic effects of PDE2 overexpression in our computational model. This limitation should be addressed in future studies developing novel computational models integrating spatial calcium handling and β-adrenoceptor and CaMKII signaling. Future work should also assess the potential antiarrhythmic effects of PDE2 overexpression in the presence of disease-related remodeling of cAMP and CaMKII signaling (e.g., in the setting of heart failure). Further studies are needed to reveal the influence of other PDE2 isoforms on the observed antiarrhythmic effects. Mitochondrial dysfunction has been shown to contribute to arrhythmia development [51]. PDE2 is the main cAMP-hydrolyzing PDE within cardiac mitochondria where it regulates respiration and permeability transition [23]. Furthermore, the PDE2A2 variant regulates the cAMP/PKA-dependent phosphorylation of dynamin-related protein 1 (Drp1) mediating mitochondrial fragmentation [24].

In conclusion, higher PDE2 abundance protects against isoprenaline-induced cardiac arrhythmia by preventing ISO-induced afterdepolarizations and spontaneous action potentials. Mechanistically, blunting of ISO-induced, Epac and CaMKII-mediated increases in I_NaL_ and SR Ca^2+^ leak, as well as reduced increase in I_CaL_ were identified as underlying mechanisms. Consequently, the increase in PDE2 found in heart disease may constitute an important cardiac defense mechanism from β-adrenergic stress. Thus, the activation of myocardial PDE2 may represent a novel intracellular anti-arrhythmic therapeutic strategy in HF.

## 4. Materials and Methods

For a detailed description of methods see Supplementary Materials.

### 4.1. Animal Care and Adult Mouse Ventricular Myocyte Isolation

The experiments comply with the ARRIVE guidelines and conform to the guidelines from Directive 2010/63/EU of the European Parliament on the protection of animals used for scientific purposes and was approved by the Dresden University Committee on the Use and Care of Animals. The PDE2 transgenic mouse line (PDE2 OE) with C57Bl/6 background exhibiting a cardiac-specific ~10 fold PDE2 overexpression under the control of the murine α-myosin heavy chain promoter was employed in this study [29,52]. The plasmid used to for PDE2A3 expression contains the complete murine α-myosin heavy chain (MHC) promoter to ensure a cardiospecific expression and the murine isoform of PDE2A3 (NCBI reference sequence: NM_001008548.3) flanked by a polyA-tail. A FVB/N strain was transduced by microinjection and resulting founder lines were crossed into a C57Bl/6 background. Adult mouse ventricular myocytes were isolated by Langendorff perfusion from PDE2 OE and wildtype (WT) littermates at 3 months as described previously [53].

### 4.2. ECG Measurements of ex vivo Perfused Hearts

A modified protocol was used to detect arrhythmia of *ex vivo* perfused hearts after reperfusion injury [54]. Briefly, mice were anaesthetized with 0.2 mL sodium pentobarbital (5% *w*/*v*). The hearts were rapidly excised and perfused with a Krebs-Henseleit buffer solution (in mM: 11.1 glucose, 2.4 CaCl_2_, 118.5 NaCl, 25 NaHCO_3_, 1.2 MgSO_4_. 1.21 NaH_2_PO_4_, 3 KCl) with physiological catecholamine concentration (10 nM norepinephrine; 3.5 nM epinephrine) at 37 °C by gravity flow in a Langendorff system. After stabilization for 15 min, left ascending coronary artery was ligated for 30 min. ECG were measured within 30 min after reperfusion (BioAmp and Powerlab system, ADInstruments). Arrhythmic events were quantified using LabChart v7. Infarct size was determined after perfusion with Evans Blue (0.5% *w/v* in PBS) (Sigma-Aldrich Chemical Co., St. Louis, MO, USA) and staining with 2,3,5-triphenyltetrazolium chloride (1% *w/v* in PBS) (TTC, Sigma-Aldrich) from 2 mm heart slices fixed in 4% formaldehyde.

### 4.3. Electrophysiology

Whole-cell voltage-clamp was used to measure L-type Ca^2+^ current (I_Ca,L_), K^+^ currents and the late Na^+^ current (I_NaL_), as described previously [55]. To assess action potential properties, APs were evoked by brief current pulses at 1 Hz. Arrhythmia provocation was initiated upon eliciting APs at 4Hz (5 s) followed by 0.125Hz (160 s) to assess the generation of arrhythmogenic early and delayed afterdepolarizations (EAD, DAD) and spontaneous action potentials (sAP). Experiments were performed at 37 °C. Cardiomyocytes were pre-incubated (10 min) in modified Tyrode solution containing the compound specified in Figure 1 and Figure 2.

### 4.4. Ca^2+^ Spark Analysis

Ca^2+^ spark measurements were performed on a laser scanning confocal microscope (LSM 5 Pascal, Zeiss). Fluorescence images of Fluo-4 AM (10 μmol/L, molecular probes) loaded ventricular myocytes were recorded in the line-scan mode after field stimulation (1 Hz).

### 4.5. Protein Quantification

Lysates of isolated cardiomyocytes were run on 10% SDS-PAGE and blotted onto nitrocellulose membranes. For protein quantification the following specific primary antibodies were used: Epac1 (cell signaling, 5D3, #4155), Epac2 (cell signaling, #4156S), CaMKIIδ total (R&D Systems, MAB4176), CaMKII pThr286 PKAIIα (Thermo, MA1-047), PKA-C (BD Bioscience, #610981), PKA IIα reg (C20) (Santa-Cruz, sc-908), PKCα (H-7) (Santa-Cruz, sc-8393), NCX1 (Swant, π 11-13), NKA (cell signaling #3010), PLM (Abcam, ab76579), EEF2 (Abcam, ab40812), GAPDH (Santa-Cruz, sc-365062).

### 4.6. In Silico Modeling

The Heijman et al. [56] model of localized cAMP/PKA signaling in the canine ventricular cardiomyocyte with the extensions proposed by Neef et al. [33] were used to investigate the effects of PDE2 overexpression. The model was implemented in Myokit [57]. Single-cell simulations were performed using the default model or in the presence of PDE2 overexpression, simulated as a two-fold increase in total PDE2 levels. Steady state properties (after pre-pacing for 2000 s) were assessed during 0.1 Hz, 1.0 Hz, or 2.0 Hz pacing with a 1.0 ms stimulus current of −80 pA/pF.

### 4.7. Statistics

Results are presented as mean ± SEM. Statistical analysis was performed with GraphPad Prism software (V7; San Diego, CA). Either unpaired Student’s *t*-tests with equal variances and normal distribution, Welch’s *t*-test with unequal variances and normal distribution or One-way ANOVA followed by Sidak multiple comparison test or a non-parametric Kruskal Wallis test followed by a Dunn’s multiple comparison test were used to determine statistical differences according to the experimental setting. *p* values of less than 0.05 were considered statistically significant.

## Data Availability

The data presented in this study are available on request from the corresponding authors.

## Supplementary Materials

The following are available online at https://www.mdpi.com/article/10.3390/ijms22094816/s1, Figure S1: PDE2 TG display diminished response of the L-type Ca^2+^ current (I_CaL_) to β-AR stimulation. Figure S2: Basal expression of protein kinases is not altered by PDE2 overexpression, Figure S3: Effects of PDE2 overexpression on downstream signaling components in the canine ventricular cardiomyocyte model by Neef et al. [33], Figure S4: Effects of PDE2 overexpression on cellular electrophysiology in the canine ventricular cardiomyocyte model by Neef et al. [33].

## Abbreviations

AAR	Area at risk
AC	Adenylyl cyclase
AIP	Autocamptide-2 Related Inhibitor Peptide
AP	Action potential
APD	Action potential duration
ATP	Adenosine triphosphate
β-AR	β-adrenoceptor
CaMKII	Ca^2+^-calmodulin-dependent protein kinase II
CaSpF	Calcium spark frequency
cAMP	3′,5′-cyclic adenosine monophosphate
cGMP	3′,5′-cyclic guanosine monophosphate
8-CPT	8-(4-chlorophenylthio)-2′-O-methyladenosine-3′,5′-cyclic monophosphate
DAD	Delayed afterdepolarization
DAG	Diacylglycerol
DRP1	Dynamin related protein 1
EAD	Early afterdepolarization
ECC	Excitation-contraction coupling
ECG	Electrocardiogram
Epac	Exchange protein directly activated by cAMP
GAF	cGMP-specific phosphodiesterases, adenylyl cyclases and FhlA
HF	Heart failure
IP_3_	Inositol triphosphate
ISO	isoprenaline
LAD	Left anterior descending coronary artery
LTCC	L-type Ca^2+^ channel
MI	Myocardial infarction
NCX	Na^+^/Ca^2+^ exchanger
NKA	Na^+^/K^+^ ATPase
NP	Natriuretic peptide
OE	Overexpression/ overexpressing
PDE	Phosphodiesterase
pGC	Particulate guanylyl cyclases
PIP_2_	Phosphatidylinositol 4,5-bisphosphate
PKA	Protein kinase A
PKC	Protein kinase C
PKI	Protein kinase inhibitor peptide
PLB	Phospholamban
PLM	Phospholemman
PVC	Premature ventricular complexes
RyR	Ryanodine receptors
sAC	Soluble adenylyl cyclases
sAP	Spontaneous action potential
SCD	Sudden cardiac death
SERCA2	SR Ca^2+^-ATPase type-2a
SNP	Sodium nitroprusside
SR	Sarcoplasmic reticulum
TTC	2,3,5-triphenyltetrazolium chloride
VES	Ventricular extrasystoles
WT	Wildtype

## References

[1] Huizar J.F., Ellenbogen K.A., Tan A.Y., Kaszala K. (2019). Arrhythmia-Induced Cardiomyopathy: JACC State-of-the-Art Review. J. Am. Coll. Cardiol..

[2] Johnson D.M., Antoons G. (2018). Arrhythmogenic Mechanisms in Heart Failure: Linking β-Adrenergic Stimulation, Stretch, and Calcium. Front. Physiol..

[3] Grandi E., Ripplinger C.M. (2019). Antiarrhythmic mechanisms of beta blocker therapy. Pharmacol. Res..

[4] Mosterd A., Hoes A.W. (2007). Clinical epidemiology of heart failure. Heart.

[5] Van Heerebeek L., Hamdani N., Falcão-Pires I., Leite-Moreira A.F., Begieneman M.P., Bronzwaer J.G., van der Velden J., Stienen G.J., Laarman G.J., Somsen A. (2012). Low Myocardial Protein Kinase G Activity in Heart Failure with Preserved Ejection Fraction. Circulation.

[6] Jhund P., Rouleau J., Swedberg K., Zile M., Lefkowitz M., Prescott M., Shi V., Solomon S., Packer M., McMurray J. (2017). Low Urinary Cgmp/Bnp Ratio Is Associated with Worse Outcomes in Heart Failure but Is Increased by Treatment with Sacubitril/Valsartan: An Analysis of Paradigm-HF. J. Am. Coll. Cardiol..

[7] McMurray J.J., Packer M., Desai A.S., Gong J., Lefkowitz M.P., Rizkala A.R., Rouleau J.L., Shi V.C., Solomon S.D., Swedberg K. (2014). Angiotensin–Neprilysin Inhibition versus Enalapril in Heart Failure. N. Engl. J. Med..

[8] Blanton R.M. (2020). cGMP Signaling and Modulation in Heart Failure. J. Cardiovasc. Pharmacol..

[9] Emdin M., Aimo A., Castiglione V., Vergaro G., Georgiopoulos G., Saccaro L.F., Lombardi C.M., Passino C., Cerbai E., Metra M. (2020). Targeting Cyclic Guanosine Monophosphate to Treat Heart Failure. J. Am. Coll. Cardiol..

[10] Bers D.M. (2002). Cardiac excitation-contraction coupling. Nature.

[11] Landstrom A.P., Dobrev D., Wehrens X.H. (2017). Calcium Signaling and Cardiac Arrhythmias. Circ. Res..

[12] Kumari N., Gaur H., Bhargava A. (2018). Cardiac voltage gated calcium channels and their regulation by β-adrenergic signaling. Life Sci..

[13] Van der Heyden M.A., Wijnhoven T.J., Opthof T. (2006). Molecular aspects of adrenergic modulation of the transient outward current. Cardiovasc. Res..

[14] Iqbal S.M., Lemmens-Gruber R. (2019). Phosphorylation of cardiac voltage-gated sodium channel: Potential players with multiple dimensions. Acta Physiol..

[15] Grimm M., Brown J.H. (2010). Beta-adrenergic receptor signaling in the heart: Role of CaMKII. J. Mol. Cell. Cardiol..

[16] Saad N.S., Elnakish M.T., Ahmed A.A., Janssen P.M. (2018). Protein Kinase A as a Promising Target for Heart Failure Drug Development. Arch. Med Res..

[17] Enriquez A., Frankel D.S., Baranchuk A. (2017). Pathophysiology of ventricular tachyarrhythmias: From automaticity to reentry. Herzschrittmacherther. Elektrophysiol..

[18] Weber S., Zeller M., Guan K., Wunder F., Wagner M., El-Armouche A. (2017). PDE2 at the crossway between cAMP and cGMP signalling in the heart. Cell. Signal..

[19] Sadek M.S., Cachorro E., El-Armouche A., Kämmerer S. (2020). Therapeutic Implications for PDE2 and cGMP/cAMP Mediated Crosstalk in Cardiovascular Diseases. Int. J. Mol. Sci..

[20] Rosman G.J., Martins T.J., Sonnenburg W.K., Beavo J.A., Ferguson K., Loughney K. (1997). Isolation and characterization of human cDNAs encoding a cGMP-stimulated 3’,5’-cyclic nucleotide phosphodiesterase. Gene.

[21] Mongillo M., Tocchetti C.G., Terrin A., Lissandron V., Cheung Y.F., Dostmann W.R., Pozzan T., Kass D.A., Paolocci N., Houslay M.D. (2006). Compartmentalized phosphodiesterase-2 activity blunts beta-adrenergic cardiac inotropy via an NO/cGMP-dependent pathway. Circ. Res..

[22] Lugnier C., Keravis T., le Bec A., Pauvert O., Proteau S., Rousseau E. (1999). Characterization of cyclic nucleotide phos-phodiesterase isoforms associated to isolated cardiac nuclei. Biochim. Biophys. Acta Gen. Subj..

[23] Liu D., Wang Z., Nicolas V., Lindner M., Mika D., Vandecasteele G., Fischmeister R., Brenner C. (2019). PDE2 regulates membrane potential, respiration and permeability transition of rodent subsarcolemmal cardiac mitochondria. Mitochondrion.

[24] Monterisi S., Lobo M.J., Livie C., Castle J.C., Weinberger M., Baillie G.S., Surdo N.C., Musheshe N., Stangherlin A., Gottlieb E. (2017). PDE2A2 regulates mitochondria morphology and apoptotic cell death via local modulation of cAMP/PKA signalling. eLife.

[25] Acin-Perez R., Russwurm M., Günnewig K., Gertz M., Zoidl G., Ramos L., Buck J., Levin L.R., Rassow J., Manfredi G. (2011). A Phosphodiesterase 2A Isoform Localized to Mitochondria Regulates Respiration. J. Biol. Chem..

[26] Mongillo M., McSorley T., Evellin S., Sood A., Lissandron V., Terrin A., Huston E., Hannawacker A., Lohse M.J., Pozzan T. (2004). Fluorescence Resonance Energy Transfer–Based Analysis of cAMP Dynamics in Live Neonatal Rat Cardiac Myocytes Reveals Distinct Functions of Compartmentalized Phosphodiesterases. Circ. Res..

[27] Sprenger J.U., Perera R.K., Steinbrecher J.H., Lehnart S.E., Maier L.S., Hasenfuss G., Nikolaev V.O. (2015). In vivo model with targeted cAMP biosensor reveals changes in receptor–microdomain communication in cardiac disease. Nat. Commun..

[28] Mehel H., Emons J., Vettel C., Wittkopper K., Seppelt D., Dewenter M., Lutz S., Sossalla S., Maier L.S., Lechene P. (2013). Phosphodiesterase-2 is up-regulated in human failing hearts and blunts beta-adrenergic responses in cardiomyocytes. J. Am. Coll. Cardiol..

[29] Vettel C., Lindner M., Dewenter M., Lorenz K., Schanbacher C., Riedel M., Lammle S., Meinecke S., Mason F.E., Sossalla S. (2017). Phosphodiesterase 2 Protects Against Catechola-mine-Induced Arrhythmia and Preserves Contractile Function After Myocardial Infarction. Circ. Res..

[30] Bers D.M., Morotti S. (2014). Ca (2+) current facilitation is CaMKII-dependent and has arrhythmogenic consequences. Front. Pharmacol..

[31] Sag C.M., Mallwitz A., Wagner S., Hartmann N., Schotola H., Fischer T.H., Ungeheuer N., Herting J., Shah A.M., Maier L.S. (2014). Enhanced late INa induces proarrhythmogenic SR Ca leak in a CaMKII-dependent manner. J. Mol. Cell. Cardiol..

[32] Métrich M., Berthouze M., Morel E., Crozatier B., Gomez A.M., Lezoualc’h F. (2010). Role of the cAMP-binding protein Epac in cardiovascular physiology and pathophysiology. Pflug. Arch. Eur. J. of Physiol..

[33] Neef S., Heijman J., Otte K., Dewenter M., Saadatmand A.R., Meyer-Roxlau S., Antos C.L., Backs J., Dobrev D., Wagner M. (2017). Chronic loss of inhibitor-1 diminishes cardiac RyR2 phosphorylation despite exaggerated CaMKII activity. Naunyn Schmiedeberg Arch. Pharmacol..

[34] Wagner S., Hacker E., Grandi E., Weber S.L., Dybkova N., Sossalla S., Sowa T., Fabritz L., Kirchhof P., Bers N.M. (2009). Ca/Calmodulin Kinase II Differentially Modulates Potassium Currents. Circ. Arrhythmia Electrophysiol..

[35] Ding B., Abe J.I., Wei H., Huang Q., Walsh R.A., Molina C.A., Zhao A., Sadoshima J., Blaxall B.C., Berk B.C. (2005). Functional role of phosphodiesterase 3 in cardiomyocyte apoptosis: Implication in heart failure. Circulation.

[36] Bristow M.R. (2011). Treatment of chronic heart failure with β-adrenergic receptor antagonists: A convergence of receptor pharmacology and clinical cardiology. Circ. Res..

[37] Holmes J.R., Kubo S.H., Cody R.J., Kligfield P. (1985). Milrinone in congestive heart failure: Observations on ambulatory ventricular arrhythmias. Am. Hear. J..

[38] Cohn J.N., Goldstein S.O., Greenberg B.H., Lorell B.H., Bourge R.C., Jaski B.E., Gottlieb S.O., McGrew I.F., Demets D.L., White B.G. (1998). A dose-dependent increase in mortality with vesnarinone among patients with severe heart failure. N. Eng. J. Med..

[39] Moreno J.D., Clancy C.E. (2012). Pathophysiology of the cardiac late Na current and its potential as a drug target. J. Mol. Cell. Cardiol..

[40] Wagner S., Maier L.S., Bers D.M. (2015). Role of sodium and calcium dysregulation in tachyarrhythmias in sudden cardiac death. Circ. Res..

[41] Shattock M.J., Ottolia M., Bers D.M., Blaustein M.P., Boguslavskyi A., Bossuyt J., Bridge J.H., Chen-Izu Y., Clancy C.E., Edwards A. (2015). Na+/Ca2+ ex-change and Na+/K+-ATPase in the heart. J. Physiol..

[42] Rivet-Bastide M., Vandecasteele G., Hatem S., Verde I., Benardeau A., Mercadier J.J., Fischmeister R. (1997). cGMP-stimulated cyclic nucleotide phosphodiesterase regulates the basal calcium current in human atrial myocytes. J. Clin. Investig..

[43] Hua R., Adamczyk A., Robbins C., Ray G., Rose R.A. (2012). Distinct Patterns of Constitutive Phosphodiesterase Activity in Mouse Sinoatrial Node and Atrial Myocardium. PLoS ONE.

[44] Galindo-Tovar A., Vargas M.L., Kaumann A.J. (2018). Phosphodiesterase PDE2 activity, increased by isoprenaline, does not reduce beta-adrenoceptor-mediated chronotropic and inotropic effects in rat heart. Naunyn Schmiedeberg Arch. Pharmacol..

[45] Vincent K.P., McCulloch A.D., Edwards A.G. (2014). Toward a hierarchy of mechanisms in CaMKII-mediated arrhythmia. Front. Pharmacol..

[46] Glynn P., Musa H., Wu X., Unudurthi S.D., Little S., Qian L., Wright P.J., Radwanski P.B., Györke S., Mohler P.J. (2015). Voltage-Gated Sodium Channel Phosphorylation at Ser571 Regulates Late Current, Arrhythmia, and Cardiac Function In Vivo. Circulation.

[47] Pereira L., Bare D.J., Galice S., Shannon T.R., Bers D.M. (2017). β-Adrenergic induced SR Ca 2+ leak is mediated by an Epac-NOS pathway. J. Mol. Cell. Cardiol..

[48] Dybkova N., Wagner S., Backs J., Hund T.J., Mohler P.J., Sowa T., Nikolaev V.O., Maier L.S. (2014). Tubulin polymerization disrupts cardiac β-adrenergic regulation of late INa. Cardiovasc. Res..

[49] Zoccarato A., Surdo N.C., Aronsen J.M., Fields L.A., Mancuso L., Dodoni G., Stangherlin A., Livie C., Jiang H., Sin Y.Y. (2015). Cardiac Hypertrophy Is Inhibited by a Local Pool of cAMP Regulated by Phosphodiesterase 2. Circ. Res..

[50] Baliga R.S., Preedy M.E.J., Dukinfield M.S., Chu S.M., Aubdool A.A., Bubb K.J., Moyes A.J., Tones M.A., Hobbs A.J. (2018). Phosphodiesterase 2 inhibition preferentially promotes NO/guanylyl cyclase/cGMP signaling to reverse the development of heart failure. Proc. Natl. Acad. Sci. USA.

[51] Yang K.-C., Bonini M.G., Dudley S.C. (2014). Mitochondria and arrhythmias. Free. Radic. Biol. Med..

[52] Subramanian H., Froese A., Jönsson P., Schmidt H., Gorelik J., Nikolaev V.O. (2018). Distinct submembrane localisation compartmentalises cardiac NPR1 and NPR2 signalling to cGMP. Nat. Commun..

[53] Börner S., Schwede F., Schlipp A., Berisha F., Calebiro D., Lohse M.J., Nikolaev V.O. (2011). FRET measurements of intra-cellular cAMP concentrations and cAMP analog permeability in intact cells. Nat. Protoc..

[54] Stables C.L., Curtis M.J. (2009). Development and characterization of a mouse in vitro model of ischaemia-induced ventricular fibrillation. Cardiovasc. Res..

[55] Foltz W.U., Wagner M., Rudakova E., Volk T. (2012). N-acetylcysteine prevents electrical remodeling and attenuates cellular hypertrophy in epicardial myocytes of rats with ascending aortic stenosis. Basic Res. Cardiol..

[56] Heijman J., Volders P.G., Westra R.L., Rudy Y. (2011). Local control of β-adrenergic stimulation: Effects on ventricular myocyte electrophysiology and Ca(2+)-transient. J. Mol. Cell. Cardiol..

[57] Clerx M., Collins P., de Lange E., Volders P.G. (2016). Myokit: A simple interface to cardiac cellular electrophysiology. Prog. Biophys. Mol. Biol..

