# Supplementary material for: Cellular Mechanisms of the Anti-Arrhythmic Effect of Cardiac PDE2 Overexpression
Source: Int J Mol Sci. 2021 May 1;22(9):4816. doi: 10.3390/ijms22094816 (PMC8125727; doi:10.3390/ijms22094816)
Supplement: Supplementary file 1 [file ijms-22-04816-s001.zip › ijms-1186328-supplementary.pdf]

Supplement

Cellular Mechanisms of the Anti-Arrhythmic Effect of Cardiac PDE2 Overexpression

Michael Wagner [1,2,†], Mirna S. Sadek [1,†], Nataliya Dybkova [3,4,†], Fleur E. Mason [3,4], Johann Klehr [1], Rebecca Firneburg [1], Eleder Cachorro [1], Kurt Richter [1,2], Erik Klapproth [1], Stephan R. Kuenzel [1], Kristina Lorenz [5,6], Jordi Heijman [7], Dobromir Dobrev [8,9,10], Ali El-Armouche [1,*], Samuel Sossalla [3,4,11,†] and Susanne Kämmerer [1,*,†]

[1] Department of Pharmacology and Toxicology, Dresden University of Technology, 01307 Dresden, Germany; michael_wagner@tu-dresden.de (M.W.); mirna.sadeks@gmail.com (M.S.S.); johann.klehr@tu-dresden.de (J.K.); rebecca.firneburg@tu-dresden.de (R.F.); eleder.cachorro_puente@tu-dresden.de (E.C.); kurt.richter@outlook.de (K.R.); erik.klapproth@tu-dresden.de (E.K.); Stephan.kuenzel@tu-dresden.de (S.R.K.)

[2] Klinik für Innere Medizin und Kardiologie, Dresden Heart Center, Dresden University of Technology, 01307 Dresden, Germany

[3] Clinic for Cardiology & Pneumology, University of Göttingen, 37075 Göttingen, Germany; ndybkov@med.uni-goettingen.de (N.D.); fleur.mason@med.uni-goettingen.de (F.E.M.); samuel.sossalla@ukr.de (S.S.)

[4] DZHK (German Centre for Cardiovascular Research), 10785 Berlin, Germany

[5] Department of Pharmacology and Toxicology, Julius-Maximilians-Universität Würzburg, 97078 Würzburg, Germany; lorenz@toxi.uni-wuerzburg.de

[6] Leibniz-Institut für Analytische Wissenschaften-ISAS-e.V., 44139 Dortmund, Germany

[7] Department of Cardiology, CARIM School for Cardiovascular Diseases, Faculty of Health, Medicine, and Life Sciences, Maastricht University, 6200 MD Maastricht, The Netherlands; Jordi.heijman@maastrichtuniversity.nl

[8] Institute of Pharmacology, West German Heart and Vascular Center, University Duisburg-Essen, 45147 Essen, Germany; Dobromir.dobrev@uk-essen.de

[9] Montréal Heart Institute, University de Montréal, Medicine and Research Center, Montréal, QC H1T 1C8, Canada

[10] Department of Molecular Physiology Biophysics, Baylor College of Medicine, Houston, TX 77030, USA

[11] Department of Internal Medicine II, University Hospital Regensburg, 93042 Regensburg, Germany

***** Correspondence: ali.el-armouche@tu-dresden.de (A.E-A.); susanne.kaemmerer@tu-dresden.de (S.K.); Tel.: +49-(0)-351-458-6300 (A.E.-A.); +49-(0)-351-458-6279 (S.K.)

† These authors contributed equally to this work.

Supplemental Figures

Figure S1

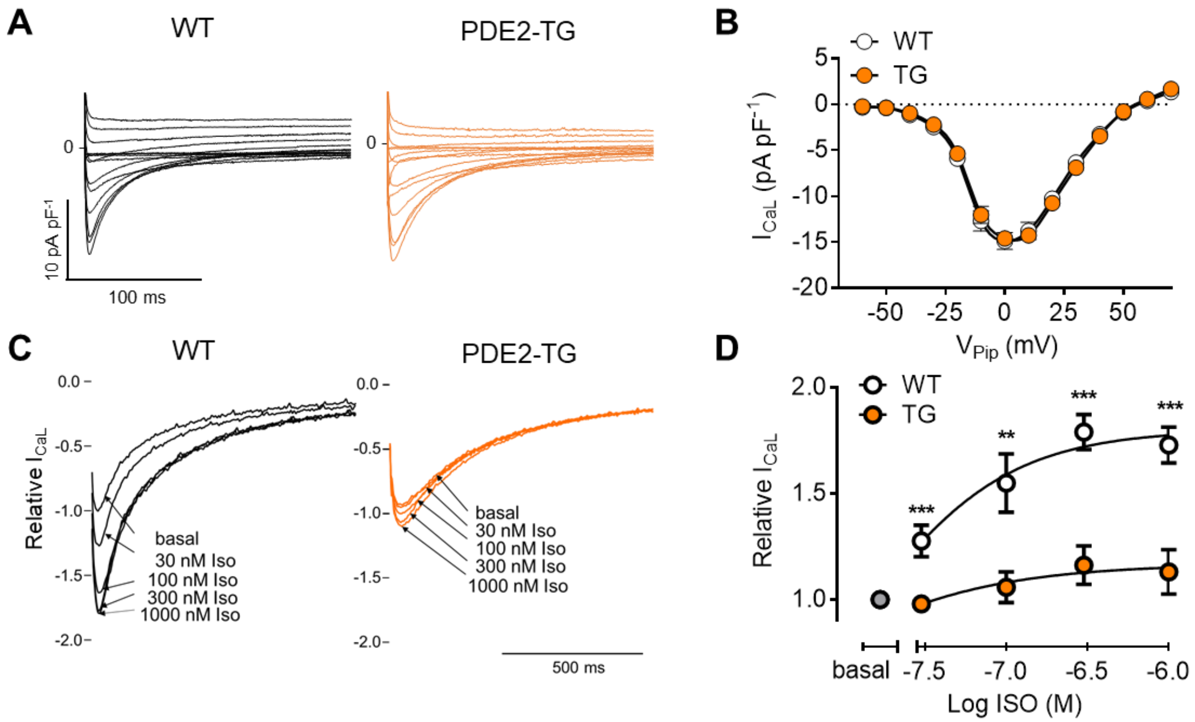

Supplemental Figure S1. PDE2 TG display diminished response of the L-type Ca^{2+} current (I_{CaL}) to β-AR stimulation. (A) Representative I_{CaL} traces under basal conditions recorded in whole-cell patch-clamp mode from WT and PDE2 TG ventricular cardiomyocytes. Cells were depolarized from -60 to 70 mV for 400 ms. (B) Average current-voltage relationship of I_{CaL} under basal conditions. Current traces were normalized to cell membrane capacitance and are presented as current densities in pA/pF. (C) Representative WT and PDE2 TG I_{CaL} traces upon cumulative β-adrenergic stimulation with isoprenaline (ISO, 0, 30, 100, 300 nM) and (D) average I_{CaL} concentration response to β-AR stimulation at 0 mV relative to its amplitude under basal conditions. **p<0.01, ***p<0.001 vs. TG (N=5 animals/genotype, 7≤n≤16 cells/group).

Figure S2

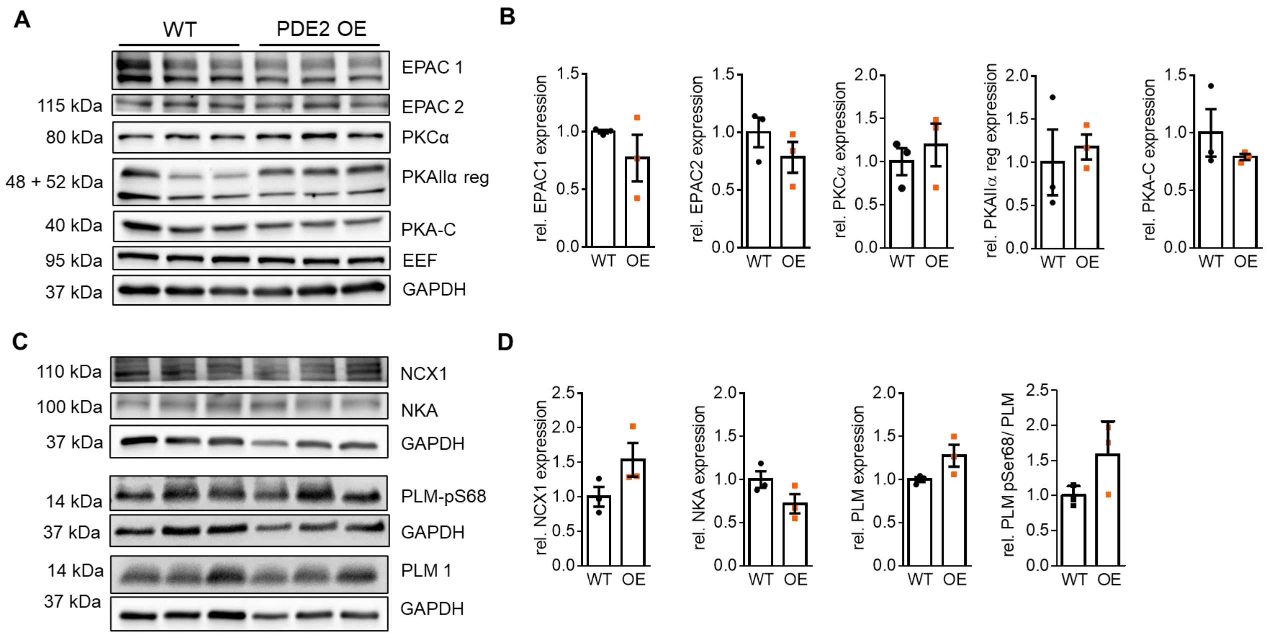

Figure S2. Basal expression of protein kinases is not altered by PDE2 overexpression. (A) Representative immunoblots and (B) quantification of protein kinase expression normalized to EEF or GAPDH, in lysates of isolated cardiomyocytes from PDE2 OE and WT mice. (N=3 animals/genotype.). (C) Representative immunoblots and (D) quantification of protein expression of Na^+/Ca^{2+} exchanger type 1 (NCX 1), Na^+/K^+ ATPase (NKA) and phospholemman (PLM) normalized to GAPDH, PLM phosphorylation at Serine 68 normalized to total PLM 1 in lysates of isolated cardiomyocytes from PDE2 OE and WT mice. (N=3 animals/genotype. *p<0.05, ***p<0.001 vs. WT).

Figure S3

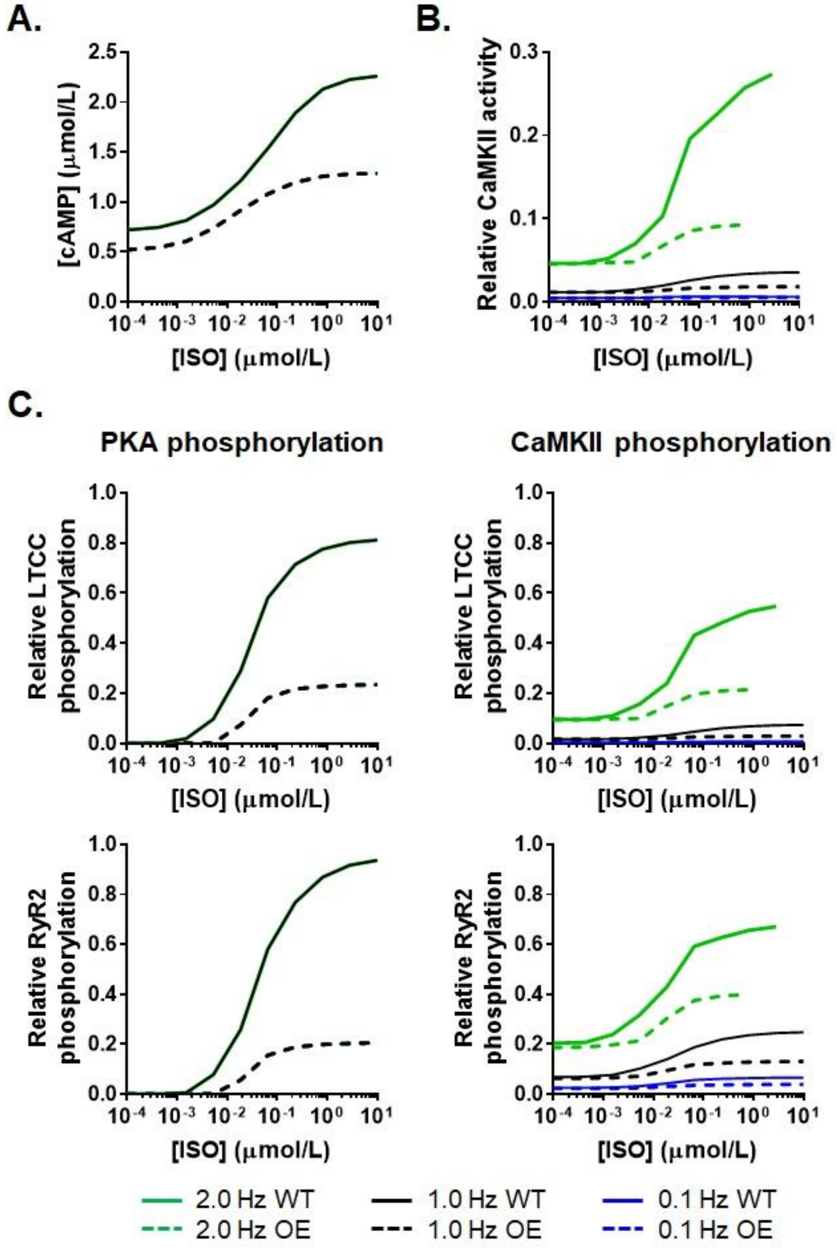

Supplemental Figure S3. Effects of PDE2 overexpression on downstream signaling components in the canine ventricular cardiomyocyte model by Neef et al. [1]. Effects of simulated two-fold transgenic PDE2 overexpression (TG; dashed lines) was compared to the default wild-type model (WT; solid lines) in the presence of various simulated isoproterenol (ISO) concentrations during 2.0, 1.0 or 0.1 Hz pacing. A. Whole-cell cAMP levels. B. Relative CaMKII activity C. PKA-dependent (left panels) and CaMKII- dependent (right panels) phosphorylation of L-type Ca2+ channels (LTCC; top panels) and ryanodine receptor channel type-2 (RyR2; bottom panels).

Figure S4

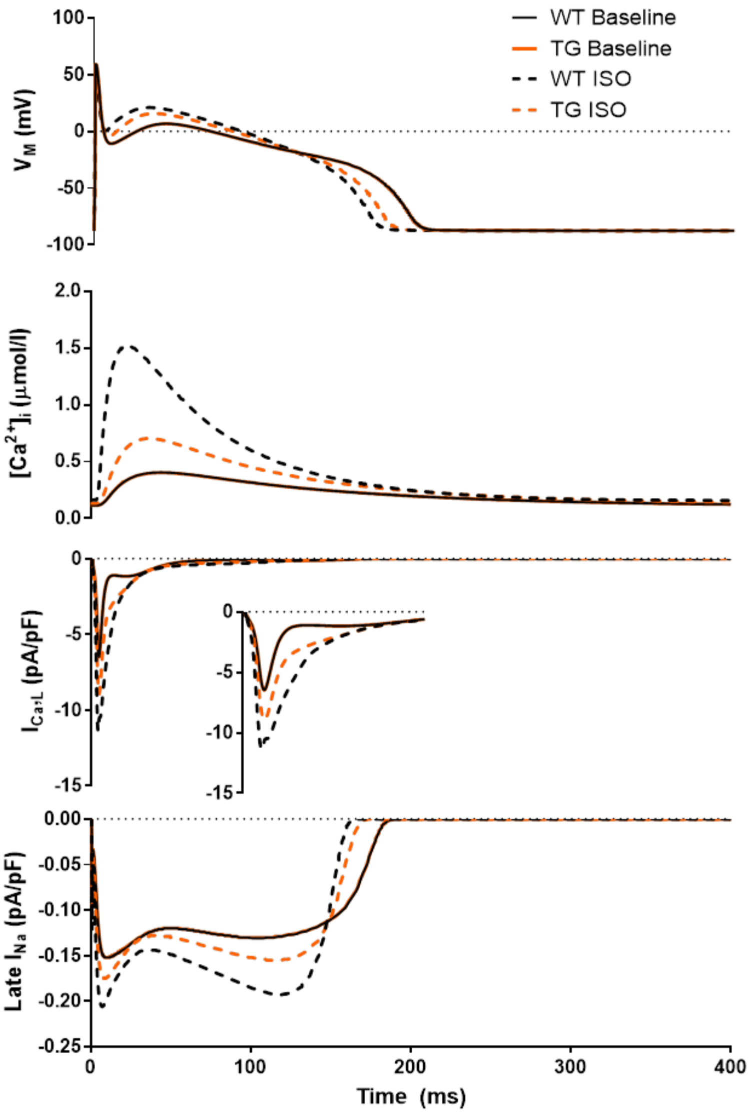

Supplemental Figure S4. Effects of PDE2 overexpression on cellular electrophysiology in the canine ventricular cardiomyocyte model by Neef et al. [1]. Steady state action potential, Ca^{2+} transient, L-type Ca^{2+} current (I_{CaL}) and late Na^+ current in the wild-type model (black lines) or model with simulated two-fold overexpression of PDE2 (orange lines) in the absence (solid lines) or presence (dashed lines) of 100 nM isoproterenol (ISO) during 2-Hz pacing. Inset shows peak I_{CaL} at an expanded scale. Note that solid black and orange lines overlap. PDE2 overexpression attenuates ISO-induced augmentation of I_{CaL} and I_{NaL}, reducing systolic Ca^{2+} transient amplitude.

Figure S5

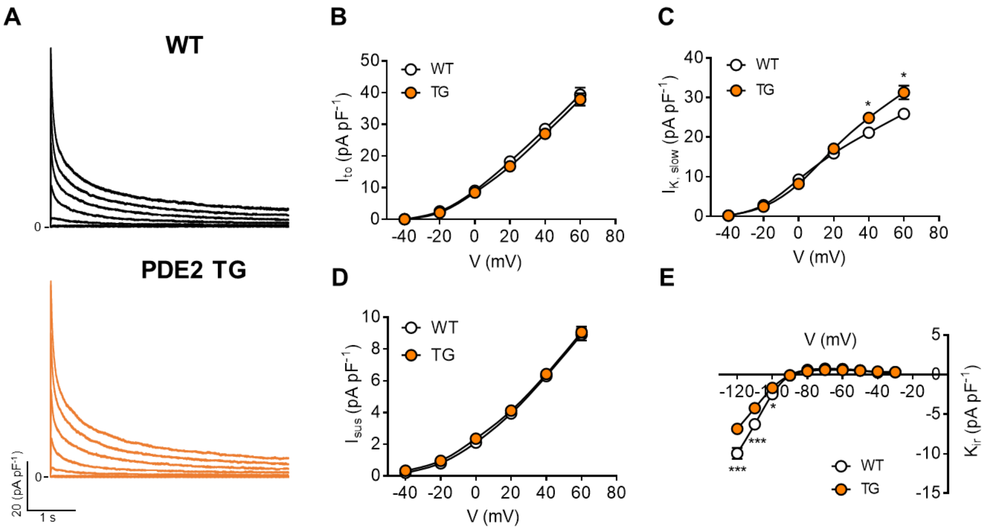

Supplemental Figure S5. PDE2 overexpression has a minor effect on K^+ currents. (A) Representative K^+ current traces recorded in whole cell patch-clamp from WT and PDE2 TG left ventricular cardiomyocytes. Average current voltage relationship of (B) transient outward current (I_{to}), (C) slowly inactivating ($I_{K,slow}$) and (D) non-inactivating sustained K^+ current component (I_{sus}) upon voltage step depolarization from -40 to 60 mV (20 mV steps). (E) Average current voltage relation of the inward rectifying (I_{Kir}) K^+ currents recorded upon voltage step hyper/depolarization from -120 to -30mV (10 mV steps). *$p<0.05$, ***$p<0.001$ (N=6 animals/genotype, $11 \leq n \leq 70$ cells/group).

Detailed Methods

All experiments were carried out according to the European Community guiding principles in the care and use of animals (2010/63/UE, 22 September 2010) and the local Institutional Animal Care and Use committees of the University of Göttingen and the Technical University of Dresden. All breeding and procedures were carried out at the animal facilities of the University of Göttingen and TU Dresden according to institutional regulations and the local guide for the use and care of laboratory animals.

Isolation of adult mouse cardiomyocytes

Ventricular myocytes were obtained from 2 to 4 months old male mice as previously described [2]. Animals were anaesthetized by intraperitoneal injection of Na^+ Thiopental 5% (8µl/g), and the heart was quickly removed and placed into cold Ca^{2+}-free PBS. The ascending aorta was cannulated and the heart was perfused (2 min) with perfusion buffer containing (in mM): NaCl 113, KCl 4.7, $MgSO_4$ 1.2, KH_2PO_4 0.6, NaH_2PO_4 0.6, $NaHCO_3$ 12, $KHCO_3$ 10, HEPES 10, D-glucose 5.5, BDM 10, taurine 30 and phenol red 0.0425. The pH was adjusted to 7.4 with KOH. For enzymatic dissociation, the heart was perfused with perfusion buffer containing Liberase^{TM} research grade (Roche Diagnostics), trypsin and 12.5 µM $CaCl_2$ for 12.5 min at 37 °C. Upon becoming flaccid, ventricular and atrial myocardium were separated. Ventricular myocardium was cut into small pieces and dispersed in solution. Ca^{2+} concentration was increased in steps every 4 min until the desired concentration was reached (32 µM to 960 µM).

Patch Clamp experiments

Action potentials, cellular arrhythmogenic events, $I_{Ca,L,}$, I_K, I_{Kir} and I_{NaL}, were measured by patch clamp technique using an EPC10 amplifier (HEKA Elektronik). Pipette resistance was 2-3 MΩ when filled with the respective internal solution. Membrane capacitance (C_m) and series resistance (R_s) were calculated and presumably compensated using the automated capacitance compensation procedure of the EPC-10 amplifier. Series resistance was in the range of ~5 MΩ, was not allowed to exceed 10 MΩ and was compensated by 85%. The reference electrode was placed in pipette solution in a separate chamber that was connected to the bath solution via an agar-agar bridge filled with pipette solution. Pipette potential (V_{Pip}) and V_m were corrected for liquid junction potentials at the bridge-bath junction. Whole-cell currents were low-pass filtered at 1 kHz and sampled at 5 kHz. Action potentials were sampled at 5 kHz. Patch clamp data were analyzed

using the PULSE-FIT software (HEKA Elektronik, Lambrecht/Pfalz, Germany), IGOR Pro (WaveMetrics, Lake Oswego, USA).

L-Type Ca^{2+} current ($I_{Ca,L}$) measurements: Pipettes were filled with an internal solution containing (in mM): CsCl 110, EGTA 10, $MgCl_2$ 5, TEA-Cl 20, HEPES 10 and Na_2-ATP 2. The pH was adjusted to 7.2 with CsOH. Extracellular Tyrode solution contained (in mM): NaCl 138, $CaCl_2$ 2, $MgCl_2$ 1, NaH_2PO_4 0.33, D-glucose 10, HEPES 10 and KCl 0 or 4. The pH was adjusted to 7.3 with NaOH. To assess current/voltage relationship in WT and PDE2-TG myocytes, cells were depolarized every 3 s, following a pre-pulse depolarization to -50 mV, from -60 to 70 mV for 600 ms and the maximal amplitude of whole-cell $I_{Ca,L}$ was measured. The use of -50 mV pre-pulse allowed the inactivation of voltage-dependent sodium currents. Cells were superfused with KCl-Ø Tyrode for 20 s prior to $I_{Ca,L}$ measurement to block K^+ currents. To assess $I_{Ca,L}$ upon β-AR stimulation, cells were superfused in bath solution with 0, 30, 100 300 nM isoprenaline for 3 min each (ISO, Sigma-Aldrich). Maximal $I_{Ca,L}$ was recorded at 0 mV for 600 ms after the pre-pulse depolarization to -50 mV. All experiments were performed at room temperature and currents were compensated for capacitance and leak currents.

K^+ current measurements: Pipettes were filled with an internal solution containing (in mM): glutamic acid 120, KCl 10, $MgCl_2$ 4, EGTA 10, HEPES 10 and Na_2-ATP 2. The pH was adjusted to 7.2 with KOH. Extracellular Tyrode solution contained (in mM): NaCl 138, KCl 4, $CaCl_2$ 2, $MgCl_2$ 1, NaH_2PO_4 0.33, D-glucose 10, HEPES 10, and Cd^{2+} 0.3 to block I_{CaL}. The pH was adjusted to 7.3 with NaOH. To elicit outward K^+ currents at room temperature, myocytes were clamped for 600 ms from the holding potential of -90 mV to test potentials between 60 mV and -80 mV in steps of 20 mV. I_{Na} was inactivated by a pre-pulse to -50 mV for 20 ms. The amplitudes of I_{to}, I_{Kslow}, and I_{sus} were determined by fitting the decay phases of the K^+ outward currents to the sum of two exponentials, as described previously [3]. K_{ir} was assessed as 2 mM Ba^{2+} sensitive current by clamping myocytes from the holding potential of -90 mV to test potentials between -120 mV and +20 mV in steps of 10 mV.

Late I_{Na} measurements: Pipettes were filled with an internal solution containing (in mM): CsCl 95, Cs-glutamate 40, NaCl 10, $MgCl_2$ 0.92, Mg-ATP 5, Li-GTP 0.3, HEPES 5, nifedipine 0.02 (to block Ca current), niflumic acid 0.03 (to block Ca-activated chloride current), 0.004 strophanthidin (to block NCX current), EGTA 1 and $CaCl_2$ 0.36 (free $[Ca^{2+}]$I, 100nM). The pH was adjusted to 7.2 with CsOH. Extracellular Tyrode solution contained (in mM): NaCl 135, TEA-Cl 5, CsCl 4, $MgCl_2$ 2, D-glucose 10 and HEPES 10. The pH was adjusted to 7.4 with CsOH. Prior to the

recordings, cells were incubated for 10 min in the bath solution containing 1 µM AIP (myristoylated, AnaSpec), 5 µM PKI (myristoylated, Tocris), 100 nM ISO, 10 µM 8-CPT (BioLog) as indicated. Myocytes were held at -120 mV at room temperature and late I_{Na} was elicited using a train of pulses to -35 mV (1000 ms duration, 10 pulses, BCL 2 s). Recordings were started 3 min after rupture. The measured current was integrated (between 100-500 ms) and normalized to the membrane capacitance.

Cellular Arrhythmia and AP assessment: The first series was detected using whole-cell patch clamp technique (Figure 1, 2). Pipettes were filled with an internal solution containing (in mM): glutamic acid 130, KCl 10, $MgCl_2$ 4, HEPES 10, Na_2ATP 2. The pH was adjusted to 7.2 with KOH. The extracellular Tyrode solution contained (in mM): NaCl 138, KCl 4, $CaCl_2$ 2, $MgCl_2$ 1, NaH_2PO_4 0.33, D-glucose 10 and HEPES 10. The pH was adjusted to 7.3 with NaOH. For the second series, the perforated patch-clamp mode was used (Figure 4). Pipettes were filled with an internal solution containing (in mM): glutamic acid 110, KCl 30, NaCl 5, $MgCl_2$ 1, HEPES 5 and amphotericin B 520 µM. The pH was adjusted to 7.2 with KOH. The extracellular Tyrode solution contained (in mM): NaCl 138, KCl 5.4, $CaCl_2$ 1, $MgCl_2$ 1, NaH_2PO_4 0.33, D-glucose 10 and HEPES 10. The pH was adjusted to 7.3 with NaOH. Cells were held at -40 mV until an access resistance below -40 MΩ was reached and incubated (10 min) in bath solution containing 100 nM ISO, 1 µM AIP or 100 nM BAY 60-7550 prior to every recording as indicated. To assess action potential properties of both WT and PDE2-TG, APs were evoked by brief current pulses of 200-1000 pA, at 1 Hz. 4 Hz stimulation (5 s), followed by 0.125 Hz (160 s) was applied to assess the generation of arrhythmogenic early and delayed afterdepolarizations (EADs, DADs) and spontaneous action potentials (sAPs). Experiments were performed at 37 °C. 1 Hz AP properties were evaluated using FitMaster V2x80 and Igor Pro 5. Quantification of cellular arrhythmogenic events was performed in cells subjected to proper 4 Hz stimulation (at least 90% of the protocol) and displaying a resting membrane potential below -75 mV, to ensure appropriate cell quality.

Measurements of SR Ca^{2+} leak and Ca^{2+} spark analysis

Ventricular myocytes were plated on laminin-coated chambers and left to settle for 15 min, followed by 15 min incubation at room temperature with Fluo-4 AM loading buffer (10 µM, Molecular Probes). Myocytes were superfused over several solution replacement steps (7-8 min per step), prior to experiment initiation, in order to remove excess Fluo-4 and to allow for adequate indicator deesterification. First solution replacement: experimental solution containing (in mM): NaCl 140, KCl 4, $MgCl_2$ 1, HEPES 5, D-glucose 10, $CaCl_2$ 2 (pH 7.4, NaOH, room temperature).

Second solution replacement: experimental solution containing either PKI (2 µM, myristoylated, Tocris) or AIP (1 µM, myristoylated, AnaSpec). Final solution replacement: experimental solution alone or containing 8-CPT (5 µM), 8-CPT (5 µM) and AIP (1µM), ISO (100 nM, Sigma), isoprenaline (100 nM) and PKI (2 µM) or isoprenaline (100 nM) and AIP (1 µM). Ca^{2+} spark measurements were carried out on a laser scanning confocal microscope (LSM 5 Pascal, Zeiss) with a 10x oil-immersion objective. Fluo-4 was excited by an argon ion laser (488 nm). Emitted fluorescence was collected through a 505 nm long-pass emission filter. Fluorescence images were recorded in the line-scan mode (width of scan line: 37.43 µm, 512 pixels per line, pixel time: 0.64 µs, number of unidirectional line scans: 10,000, measurement period: 7.68 s). Confocal line scans were performed at rest after a brief period of field stimulation to load the SR (10 pulses, 1 Hz, 20 V). Experiments were performed at 37 °C. Ca^{2+} sparks were analyzed in SparkMaster for ImageJ. Mean spark frequency (CaSpF) was normalized to cell width and scan rate (100 $µm^{-1}*s^{-1}$). Mean Ca^{2+} leak per myocyte was calculated by multiplying spark frequency with spark amplitude (F/F_0), spark width (µm) and spark duration (ms). In myocytes displaying pro-arrhythmic activity (Ca^{2+} waves or spontaneous transients) Ca^{2+} sparks were analysed up until the first pro-arrhythmic event.

Protein isolation and immunoblot

Isolated cells pellets were homogenized in RIPA lysis buffer containing 150 mM NaCl, 50 mM Tris-HCl, 1 mM EDTA, 1% IGEPAL CA-630, 0.25% Na-deoxycholate, 0.1% SDS and protease inhibitors (Merck PhosSTOP™, Merck cOmplete™ Mini) by mechanical disruption (QIAGEN TissueLyser LT) for 4 min at 50 Hz and centrifuged (4 °C, 5.000 xg, 15 min). For immunoblot, total protein extracts (20 µg) were run on a 10% SDS-PAGE (EPAC1, EPAC2, CaMKIIδ, CaMKII pThr286, PKCα, PKAIIαreg, PKA-C, EEF2, GAPDH) resp. Tris/Tricine gel (NCX1, NKA, PLM, PLM pSer68, GAPDH) and blotted to nitrocellulose membrane. Successful transfer was proven by Ponceau S staining. After blocking the membranes with 5% BSA (CaMKII pThr286, PLM pSer68), resp. 5% milk buffer (EPAC1, EPAC2, CaMKIIδ, PKCα, PKAII αreg, PKA-C, NCX1, NKA, PLM, EEF2, GAPDH) for 1 h at room temperature, membranes were probed with primary antibodies (for dilutions see table below) overnight at 4 °C.

antibody	dilution	dilution buffer
EPAC1, Cell Signaling #4155	1:1.000	0.1 % TBS-T
EPAC2, Cell Signaling, #41565	1:1.000	0.1 % TBS-T
CaMKII delta total, R&D, MAB4176	1:200	0.1 % TBS-T
CaMKII pThr286, Thermo, MA1-047	1:1.000	0.1 % TBS-T
PKCα, Santa cruz, sc-8393	1:500	0.1 % TBS-T

PKAII αreg, Santa cruz, sc-908	1:500	5 % milk buffer
PKA-C, BD, #610981	1:3.000	0.1 % TBS-T
NCX1, Swant, π 11-13	1:500	0.1 % TBS-T
NKA, Cell Signalling, #3010	1:1.000	0.1 % TBS-T
PLM, Abcam, A0545	1:1.000	0.1 % TBS-T
PLM pS68, Abnova, MAB0116	1:200	0.1 % TBS-T
EEF2, Abcam, ab40812	1:50.000	0.1 % TBS-T
GAPDH, Santa cruz, sc-365062	1:1.000	0.1 % TBS-T

After washing, membranes were incubated with the secondary antibody (anti-mouse: Sigma A3683, resp. anti-rabbit: Sigma A0545; each diluted in 5% milk buffer) for 1 h at room temperature. After final washing, membranes were incubated with ECL substrate (SuperSignal^{TM} West Femto Maximum Sensitivity Substrate, resp. SuperSignal^{TM} West Dura Extended Duration Substrate) and images were acquired using Fusion FX chemiluminescence imaging system. Densitometric analysis was performed using the FusionCapt Advance software.

ECG measurements of *ex vivo* Langendorff perfused hearts

The procedure was followed with some modifications to isolated murine hearts, as described [4]. C57BL/6J wild-type mice (11-13 weeks old) were anesthetized with 0.2-0.4 ml Na^+ Thiopental (5% w/v) and hearts were then rapidly excised and placed in ice-cold Ca^{2+}-free PBS. Following aorta cannulation, hearts were perfused with Ca^{2+}-free PBS to remove blood and a silk 6-0 suture was sewn loosely and threaded around the left anterior descending coronary artery (LAD), 2-3 mm distal from the left atrial appendage. Cannulated hearts were then retrogradely perfused by gravity flow on a Langendorff perfusion system at 37 °C with a Krebs-Henseleit buffer solution (in mM: D-glucose 11.1, $CaCl_2$ 2.4, NaCl 118.5, $NaHCO_3$ 25, $MgSO_4$ 1.2. NaH_2PO_4 1.21, KCl 3) with physiological catecholamine concentration (10 nM norepinephrine; 3.5 nM epinephrine) [5]. After stabilization for 15 min, ischemia was induced by ligating the left anterior descending coronary artery (LAD) placing a PE10 tubin in the transient suture and tightening it, so that the tubin was tightly pressed against the LAD, stopping the flow. After 30 min, the tube was removed to allow reperfusion for another 30 minutes. A bipolar electrocardiogram was recorded at 1 kHz with an ECG (BioAmp and Powerlab system, ADInstruments) connected to a PC with LabChart (version 7). The positive electrode was connected close to the right atrial appendage and the negative electrode to the left ventricle.

Infarct size was determined as previously described [6]. Briefly, after 30 min reperfusion, hearts were removed from the Langendorff system and the tube was tightly pressed against the LAD

again. Hearts were then infused with Evans blue (0.5% w/v in PBS) (Sigma-Aldrich) via the cannula into the coronary system and wrapped in cling film. After 15 min at -20 °C, the tube was removed and the hearts were decannulated and sectioned in 2 mm thick slices. Each slice was placed in a 2 ml Eppendorf vial and immersed in 2,3,5-triphenyltetrazolium chloride (1% w/v in PBS) (TTC, Sigma-Aldrich). To avoid hearts sections curling on themselves, they were first incubated for 5 min at room temperature and then incubated in a thermomixer at 37 °C and agitated at 350 rpm for 15 min. To enhance staining contrast, sections were fixed in 4% formaldehyde for 90 min. Hearts sections were finally photographed with a dissection microscope (Olympus SZ61) with adjustable fibre optic light source (Olympus KL1500 LCD) and attached digital camera (Leica MC170 HD). Infarct quantification was performed on digital photographs by manually contouring the differentially coloured left ventricle subsets (remote myocardium = blue, area at risk (AAR) = non-blue, and area of necrosis (AON) = white) using the image analysis software ImageJ (NIH, Bethesda, MD). The relative areas of these subsets were obtained using a pixel count tool and a relative AON to AAR percentage was calculated.

References

1. Neef, S.; Heijman, J.; Otte, K.; Dewenter, M.; Saadatmand, A. R.; Meyer-Roxlau, S.; Antos, C. L.; Backs, J.; Dobrev, D.; Wagner, M.; Maier, L. S.; El-Armouche, A., Chronic loss of inhibitor-1 diminishes cardiac RyR2 phosphorylation despite exaggerated CaMKII activity. *Naunyn-Schmiedeberg's archives of pharmacology* **2017,** 390, (8), 857-862.
2. Börner, S.; Schwede, F.; Schlipp, A.; Berisha, F.; Calebiro, D.; Lohse, M. J.; Nikolaev, V. O., FRET measurements of intracellular cAMP concentrations and cAMP analog permeability in intact cells. *Nature protocols* **2011,** 6, (4), 427-38.
3. Xu, H.; Guo, W.; Nerbonne, J. M., Four kinetically distinct depolarization-activated K+ currents in adult mouse ventricular myocytes. *The Journal of general physiology* **1999,** 113, (5), 661-78.
4. Stables, C. L.; Curtis, M. J., Development and characterization of a mouse in vitro model of ischaemia-induced ventricular fibrillation. *Cardiovascular research* **2009,** 83, (2), 397-404.
5. Grouzmann, E.; Cavadas, C.; Grand, D.; Moratel, M.; Aubert, J. F.; Brunner, H. R.; Mazzolai, L., Blood sampling methodology is crucial for precise measurement of plasma catecholamines concentrations in mice. *Pflugers Archiv : European journal of physiology* **2003,** 447, (2), 254-8.
6. Bohl, S.; Medway, D. J.; Schulz-Menger, J.; Schneider, J. E.; Neubauer, S.; Lygate, C. A., Refined approach for quantification of in vivo ischemia-reperfusion injury in the mouse heart. *American journal of physiology. Heart and circulatory physiology* **2009,** 297, (6), H2054-8.
